# Neuron‐Inspired Steiner Tree Networks for 3D Low‐Density Metastructures

**DOI:** 10.1002/advs.202100141

**Published:** 2021-08-11

**Authors:** Haoyi Yu, Qiming Zhang, Benjamin P. Cumming, Elena Goi, Jared H. Cole, Haitao Luan, Xi Chen, Min Gu

**Affiliations:** ^1^ Institute of Photonic Chips University of Shanghai for Science and Technology Shanghai 200093 China; ^2^ Centre for Artificial‐Intelligence Nanophotonics School of Optical‐Electrical and Computer Engineering University of Shanghai for Science and Technology Shanghai 200093 China; ^3^ Laboratory of Artificial‐Intelligence Nanophotonics School of Science RMIT University Melbourne VIC 3001 Australia; ^4^ Chemical and Quantum Physics School of Science RMIT University Melbourne VIC 3001 Australia

**Keywords:** biomimetic, low‐density metamaterials, path optimization, Steiner tree problem, 3D two‐photon nanolithography, topological photonics

## Abstract

Three‐dimensional (3D) micro‐and nanostructures have played an important role in topological photonics, microfluidics, acoustic, and mechanical engineering. Incorporating biomimetic geometries into the design of metastructures has created low‐density metamaterials with extraordinary physical and photonic properties. However, the use of surface‐based biomimetic geometries restricts the freedom to tune the relative density, mechanical strength, and topological phase. The Steiner tree method inspired by the feature of the shortest connection distance in biological neural networks is applied, to create 3D metastructures and, through two‐photon nanolithography, neuron‐inspired 3D structures with nanoscale features are successfully achieved. Two solutions are presented to the 3D Steiner tree problem: the Steiner tree networks (STNs) and the twisted Steiner tree networks (T‐STNs). STNs and T‐STNs possess a lower density than surface‐based metamaterials and that T‐STNs have Young's modulus enhanced by 20% than the STNs. Through the analysis of the space groups and symmetries, a topological nontrivial Dirac‐like conical dispersion in the T‐STNs is predicted, and the results are based on calculations with true predictive power and readily realizable from microwave to optical frequencies. The neuron‐inspired 3D metastructures opens a new space for designing low‐density metamaterials and topological photonics with extraordinary properties triggered by a twisting degree‐of‐freedom.

## Graphical abstract

Neuron‐inspired Steiner tree networks are proposed and studied in this work as a new method to create low‐density metamaterials, where novel mechanical properties and photonic topological quasiparticles can be discovered. The 8‐points Euclidean Steiner tree networks not only demonstrate a twist‐induced mechanical property enhancement but also host an exotic photonic Fermion resulting from the accidental degeneracy.

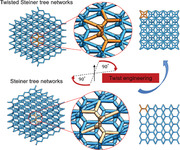

## 1 Introduction

Three‐dimensional (3D) metamaterials are artificially manufactured composite materials that derive their extraordinary properties from internal structures.^[^ ^1^, ^2^, ^3^, ^4^ ^]^ Incorporating biomimetic geometries, such as honeycomb from beehives, gyroid from butterfly wings,^[^ ^5^, ^6^ ^]^ and Kelvin foam from bubble systems,^[^ ^7^ ^]^ into the design and fabrication of micrometer and nanometer scale metastructures has led to the creation of various types of low‐density metamaterials with peculiar properties, such as ultralightweight,^[^ ^8^ ^]^ ultrastiffness,^[^ ^9^, ^10^ ^]^ and photonic/phononic phenomena including circular dichroism and optical activity.^[^ ^11^, ^12^, ^13^ ^]^ These low‐density metamaterials, whether mechanically soft or hard,^[^ ^14^ ^]^ are greatly valued for engineering applications,^[^ ^15^ ^]^ including thermal insulation, shock or vibration damping, and catalyst support, but also have become a fertile ground for the discovery of conventional (Majorana, Weyl, and Dirac) and unconventional quasiparticles (“exotic fermions”) in topological photonics.^[^ ^16^, ^17^, ^18^ ^]^

The traditional approach to design low‐density metamaterials is based on the concept of “minimal surfaces,” in particular biomimetic minimal surfaces.^[^ ^19^ ^]^ Various types of low‐density metamaterials have been created and studied, such as the Kelvin foam lattice, the octahedron trusses,^[^ ^20^ ^]^ the gyroid lattice,^[^ ^21^, ^22^ ^]^ and the shellular.^[^ ^23^ ^]^ The common driving force behind the prevalence of minimal surface‐based solutions in nature is the fact that natural structures are “programmed” to fill and stabilize a volume with minimum energy expenditure.^[^ ^24^ ^]^ In particular, these structures possess fascinating symmetries of certain crystallographic groups,^[^ ^25^ ^]^ where novel properties could be derived. For example, gyroids, the infinitely connected triply periodic minimal surfaces discovered by Alan Schoen in 1970,^[^ ^5^ ^]^ have been widely found in butterfly wings and in certain surfactant or lipid mesophases, the space group of which is I4_1_32 (no. 214).^[^ ^26^ ^]^ The gyroids were not only studied as a mechanical metamaterial,^[^ ^27^ ^]^ but also were discovered to host Weyl points and exhibit topological circular dichroism when a symmetry break is introduced.^[^ ^28^ ^]^ However, filling a volume by minimizing surface area to create metastructures is not the only natural imperative at play, and other opportunities to mimic the solutions of nature are yet to be exploited.

In this work, we emulate the structural features of biological neural networks (BNNs) to create 3D low‐density metamaterials interconnecting a volume by means of the shortest connection distance: a biological solution to the so‐called Steiner tree problem.^[^ ^29^ ^]^ The resulting 3D Steiner tree networks (STNs) can be fabricated as metamaterials with nanometric feature sizes by using two‐photon nanolithography (TPN) technology, which exhibits remarkable mechanical and topological properties triggered by a twisting degree‐of‐freedom. We show that a twist of 90° in 3D STNs leads to a more even redistribution of the strain energy between the top layer and the bottom layer along the z‐direction in the unit cell of T‐STNs, resulting in an enhanced Young's modulus and yield strength at different mass density scales (5.05%–14.84% of the bulk material, with the smallest feature size around 200 nm). Interestingly, this twisting geometry change in the STNs also gives rise to a topological transition due to the strong coupling between the eigenmodes in different photonic bands perpendicular to the z‐direction (the k*_x_*−k*_y_* plane in Brillouin zone) through accidental degeneracy, where a Dirac‐like conical dispersion is formed (a triple bands degeneracy) at the center of Brillouin zone,^[^ ^30^, ^31^ ^]^ carrying a topological charge of −2, the emergence of which can be related to the “twisted magic angle” phenomenon recently found in twistronics, such as bilayer graphene and 2D van der Waals materials.^[^ ^32^, ^33^, ^34^ ^]^ The 3D STNs derived from this novel Steiner tree path‐optimization approach open a new space for the realization of novel 3D low‐density metamaterials and a novel platform to study 3D topological photonics. These advances can enable applications in mechanically robust optical interfaces, such as 3D dielectric materials with zero refractive index,^[^ ^35^ ^]^ and may also provide a new platform for photonic neural networks with higher degrees of connectivity.^[^ ^36^, ^37^ ^]^

## 2 Steiner Tree Geometry

Neurons pursue the “**shortest connection distance**” for signal transmission and optimization.^[^ ^38^ ^]^ This characteristic corresponds to the “Steiner tree problem” proposed by Jakob Steiner in the 1880s,^[^ ^39^ ^]^ which focuses on finding the shortest connection distance for a given number of sites in 2D or 3D space. The Steiner tree problem has been widely used to find solutions for the global positioning system routing, infrastructure design, and computer chip design,^[^ ^40^ ^]^ but has never been considered as a physical geometry in 3D space.

**Figure 1:**
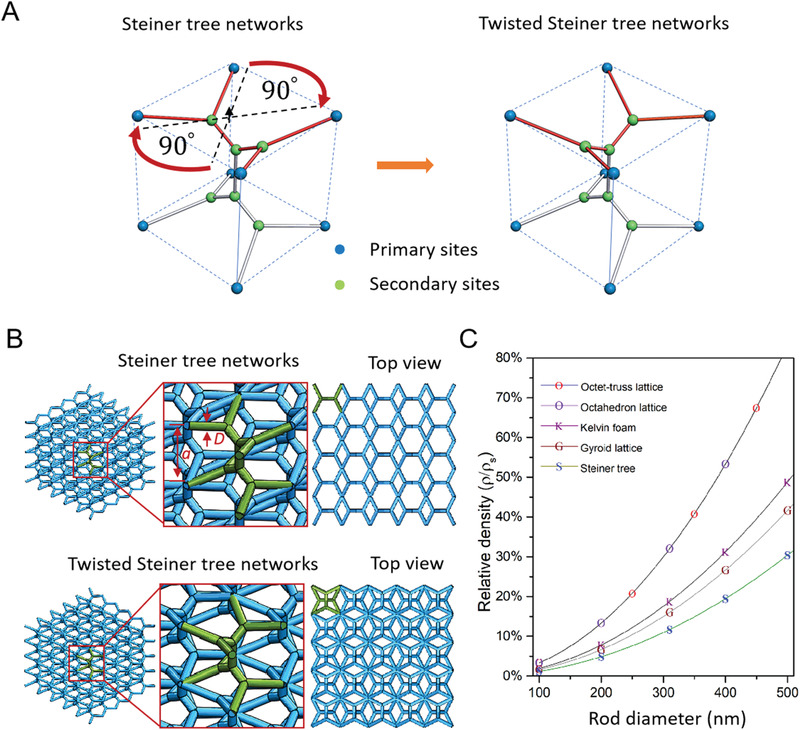
Neuron‐inspired Steiner tree networks. a) STNs (left) and T‐STNs (right) are formed from path optimization by introducing secondary sites (green spheres) to connect primary sites (blue spheres) to achieve the shortest connection distance in a simple cubic Bravais lattice. b) Reconstruction of solid STNs and T‐STNs using a cylindrical expansion into rods. The low‐density meta‐structures are constructed by moving the unit cell of STNs and Twisted STNs along x, y, and z directions, unit cells are highlighted in green. c) Comparison of the relative density between traditional network structures derived from triply periodic minimal surface (TPMS) and neuron inspired STNs (all unit cell sizes are fixed to be 2 µm).

The 3D STNs we present here are 8‐point Euclidean STNs achieved using a branching axon and dendrite optimization approach (Discussed in the Supporting information Section S1).^[^ ^41^ ^]^ In this path‐optimization approach, eight primary network sites (blue spheres shown in **Figure** **1**a–c) starting from a primitive simple cubic Bravais lattice are allowed to connect to each other through dendritic connections at secondary networks sites (green spheres),^[^ ^42^ ^]^ similar to dendritic synaptic connections of a biological neural network.

The solution of this optimization with the shortest path length and lowest symmetry is shown in Figure 1a (left). We refer to this solution as a “STNs,” which consists of six dendritic triple‐junctions (with a joint angle of 120°) connecting the eight primary network sites by 14 individual paths. The STNs prefer paths along a single lattice direction, yielding a structure within the orthorhombic space group P*mmm* (no. 47) which has mirror symmetry (and by consequence twofold rotational symmetries) along each lattice direction.^[^ ^43^ ^]^ However, this low symmetry solution can be improved upon by carefully engineering of the STNs connections. In Figure 1a (right), we create a second solution to the Steiner tree problem by splitting the STNs at the (002) plane and applying a deliberate 90° twist to one half around the [001] axis. We refer to this second unique solution of equal lowest path length as a “twisted STNs (T‐STNs)” which consists of the same six dendritic triple‐junctions, three of which are rotated by 90° to form a structure in the tetragonal space group $P\bar{4}m2$ (no. 115). This space group possesses a higher degree of symmetry, specifically the fourfold improper rotational symmetry along the principal axis and two mirror symmetries along the secondary and tertiary directions. These changes to the symmetry improve both the mechanical strength of the STNs and provide a means to realize novel topological properties as detailed in Sections 3 and 4.

For the sake of brevity, we limit our discussion in this work to 3D STNs optimized from the 3D primitive simple cubic configuration, as its 2D counterpart (simple cubic symmetry) was widely studied in metamaterials.^[^ ^30^ ^]^ Optimization on other 3D Bravais lattices or with more primary sites may give rise to more 3D STNs with unique properties and should be the subject of future investigations.

### 2.1 Nanolithographic Realization of 3D STNs

**Figure 2:**
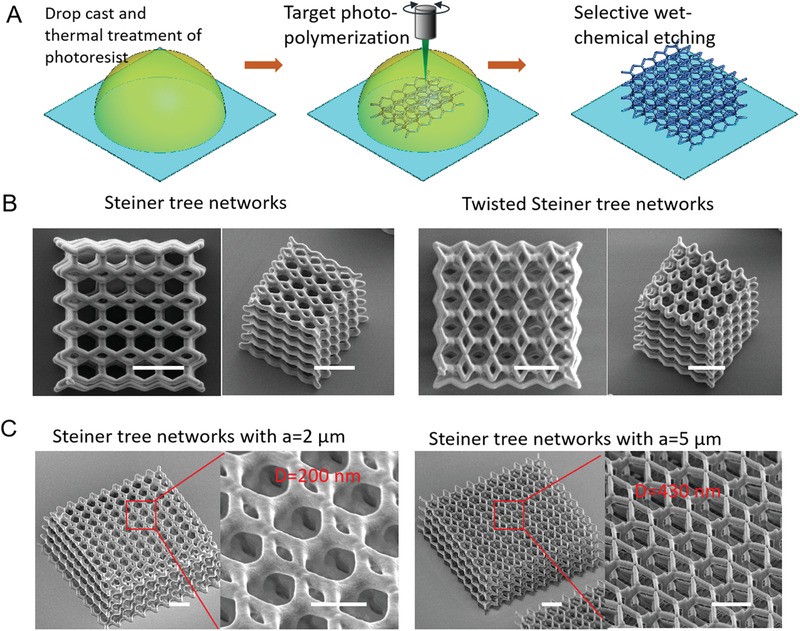
3D Two‐photon nanolithography fabrication of 3D STNs. a) Process of a typical two‐photon nanolithography technique. b) Scanning electron microscope (SEM) images of fabricated STNs and T‐STNs nanostructures with a unit size of 2 µm and a rod diameter of 200 nm for mechanical measurement, and the scale bars are 4 µm. A complete range of STNs nanostructures with varied rod sizes are shown in Figure S2 (Supporting Information). c) SEM images of Steiner tree networks with varied periodicities and rod sizes, scale bars are 2 µm (left) and 5 µm (right).

The solutions to the 3D Steiner tree problem are mathematical constructs with no volume and hence pose fabrication challenges similar to those of fabricating zero‐thickness minimal surfaces. To solve this challenge and create 3D STNs for the first time, we employ galvo‐dithered TPN (GD‐TPN). GD‐TPN, illustrated in **Figure** **2**a and detailed in Section S3 in the Supporting Information, enables the paths of mathematical networks to be traced out in 3D space while preserving their symmetry: a critical requirement if their mechanical and topological properties are to be preserved. This physical realization of 3D STNs with GD‐TPN results in a cylindrical expansion of the network segments into rods with a circular diameter *D* in a unit cell with the size of *a* as shown in Figure 1b (the realization process is shown in Movie 1, Supporting Information).

### 2.2 Relative Density of 3D STNs

The material efficiency of 3D STNs can be evaluated by analyzing the relative density of the network. In the cylindrical expansion scenario of GD‐TPN, the analytical expression for the relative density of a 3D STNs can be defined as the total volume of the rod material relative to the total volume of the unit cell. The relative density of 3D STNs can therefore be written as

(1) $$\bar{\rho }=\left(\frac{3\sqrt{3}+1}{4}\right)\;\pi \times \frac{D^{2}}{a^{2}}\times \rho_{s}$$

in which *ρ*_s_ is the mass density of the bulk material.

Compared to other widely studied lattice structures with an identical rod diameter and unit cell size,^[^ ^18^ ^]^ the smallest relative density of neuron‐inspired 3D STNs is ensured by the path‐optimization algorithm, as shown in Figure 1c. The 3D STNs have up to 3 times lower relative density compared to the gyroid network and are up to 5 times lower density than the octahedron network. This low‐density feature of 3D STNs is very advantageous for potential applications requiring minimal material use, such as cell scaffolds for biomedical engineering, shock damping, and thermal insulators. Both the STNs and T‐STNs have equal relative density due to their equal minimal path length. However, they differ markedly in their mechanical properties and topological properties.

## 3 Twist‐induced Enhancement of Mechanical Strength

A key desire in the search for low‐density metamaterials is a lower relative density while maintaining high load‐bearing capability. Rigidity analysis of 3D STNs based on Maxwell's stability criterion predicts that 3D STNs are ideal nonrigid bending‐dominated networks, similar to gyroids and kelvin foam lattices (see Table S1, Supporting Information),^[^ ^44^ ^]^ the properties of which scale around $E\approx E_{s}{\bar{\rho }}^{2}$ and $\sigma \approx \sigma_{s}{\bar{\rho }}^{2}$, where *E*_s_ and *σ*_s_ are the Young's modulus and yield strength of the bulk material. Here we demonstrate that the mechanical strength of STNs is not only comparable to traditional bending‐dominated networks with lower relative densities but that by twisting the STNs into T‐STNs we can induce an enhancement in both the Young's modulus and yield strength.

We evaluated the mechanical properties of 3D STNs both numerically using finite element method (FEM) and experimentally using the TPN technique and nanoindentation. For the experimental characterization, 3D STNs were fabricated with a volume of 5 × 5 × 5‐unit cells at a unit cell size of 2 µm. Two examples with rod diameters around 200 nm are shown in Figure 2b. By tuning the fabrication conditions, 3D STNs with rod diameters ranging from 200 to 460 nm and relative densities ranging from 5.05% to 14.84% were fabricated with high integrity as shown in Figure S3 (Supporting Information).

**Figure 3:**
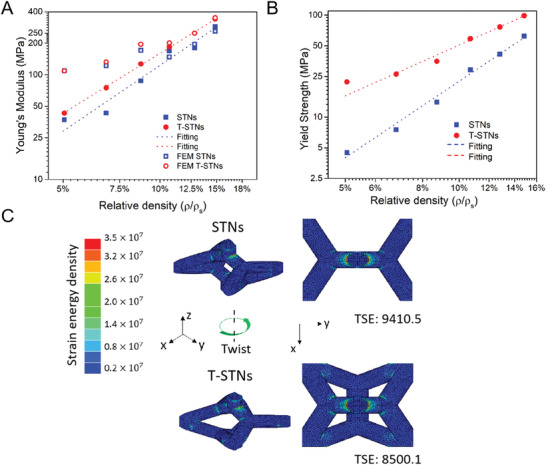
Twist induced mechanical enhancement of STNs and T‐STNs. a) Simulated and measured relative Young's modulus as a function of relative density. b) Measured relative yield strength as a function of relative density. Both figure a) and b) are plotted in logarithmic coordinates (detailed data are shown in Tables S3 and S4 (Supporting Information)). c) Simulation of the strain energy density within the STNs with a rod diameter 0.2*a* when subject to a uniaxial compression along the z‐direction. The total strain energy (TSE) is the sum of all strain energy within the structure.

The mechanical strength of the fabricated 3D STNs was measured using monotonic compression experiments on a Hysitron TI 950 TriboIndenter (see Section S4 in the Supporting Information for detailed discussion and a complete set of measurement data in Figures S7 and S8, Supporting Information). **Figure** **3**a,b shows the experimental Young's modulus and yield strength of the STNs and T‐STNs as a function of relative density. The Young's modulus of both the STNs and T‐STNs is found to follow a power law relationship that is also observed in traditional low‐density metamaterials, such as the Gyroid lattice and Kelvin foam. The resulting Young‘s modulus curves for STNs and T‐STNs are

(2) $$E_{\mathrm{STNs}}=1.508\times E_{s}{\bar{\rho }}^{2.089}$$

(3) $$E_{T-\mathrm{STNs}}=1.290\times E_{s}{\bar{\rho }}^{1.903}$$

Similarly, the yield strength of both structures also scales with a power law dependence with resulting relationships to the relative density of

(4) $$\sigma_{\mathrm{STNs}}=1.299\times \sigma_{s}{\bar{\rho }}^{1.351}$$

(5) $$\sigma_{T-\mathrm{STNs}}=2.308\times \sigma_{s}{\bar{\rho }}^{1.656}$$

Interestingly, there is a noticeable increase in both the Young's modulus and the yield strength between STNs and T‐STNs. The Young's modulus of the T‐STNs are on average 20% higher than those of STNs. At a relative density of 5.04%, the Young's modulus of STNs is around 37.1 MPa, while the T‐STNs have a strength of 43.4 MPa. This increase in Young's modulus is more observable when the relative density increases, and a similar phenomenon is observed in the yield strength of the STNs (shown in Figure 3b).

The numerical FEM simulations of the Young's modulus of the STNs (shown in Figure 3a) predict a similar enhancement to the mechanical strength of STNs and Twisted STNs at different relative density scales. However, as the FEM model is not able to account for the contribution of the joints between the rods to the overall mechanical properties, which is explained by Timoshenko solid‐rod theory,^[^ ^45^ ^]^ there are slight differences between the FEM simulation and experimental results at low relative densities (<10%). Complete analysis of this phenomenon requires in‐depth investigations into the role of joints on the mechanical properties, which is outside the scope of this work.

The role of the engineered twist operation in the improvement of mechanical strength can be understood by analyzing the strain energy density within the loaded networks. The strain energy density for each case was calculated using FEM, and the definition of strain energy is $\varphi \;=\frac{1}{2}\;\frac{V}{E}\;\sigma^{2}$, where *V* is the volume of the constituent material and *σ* is the applied stress. Figure 3c shows the calculated strain energy for STNs and T‐STNs with equal volume and when subject to identical stress. The total strain energy imparted onto the T‐STNs structure is lower than that for the STNs indicating an ability to withstand greater stress and stronger mechanical strength. The origin of this improvement lies in the uniform distribution of load‐carrying capacity in the T‐STNs. Specifically, the twist change in T‐STNs results in a redistribution of the strain energy between the top layer and the bottom layer of the unit cell along the z‐direction (shown in Figure 3c), therefore increases the Young's modulus and yield strength along the z‐direction compared with STNs which can be explained using optimal isotropic stiffness theory (see Section S5 for detailed simulation setup in the Supporting Information).^[^ ^46^ ^]^

## 4 Twist‐induced Topological Transition

Apart from the twist‐induced mechanical enhancement in STNs, the engineered twist also results in an atypical transition of the topology of photonic band structures from STNs to T‐STNs. This transition in T‐STNs gives rise to the emergence of an “erotic fermion” (a Dirac‐like triply degenerate point, which we use “triple point” for abbreviation).^[^ ^15^ ^]^ The triple point found here is different from the widely studied Dirac points and Weyl points, despite that both Dirac and Dirac‐like points exhibit linear dispersion. The triple point in STNs metamaterial emerges due to the twist‐induced accidental degeneracy,^[^ ^47^ ^]^ carrying a topological charge of −2. This is out of the traditional definition of fermions (such as Majorana, Weyl, and Dirac, which are constrained by Poincare symmetry) in topological semimetals,^[^ ^19^ ^]^ and therefore making T‐STNs a topological nontrivial material to study novel topologies in photonics.

**Figure 4:**
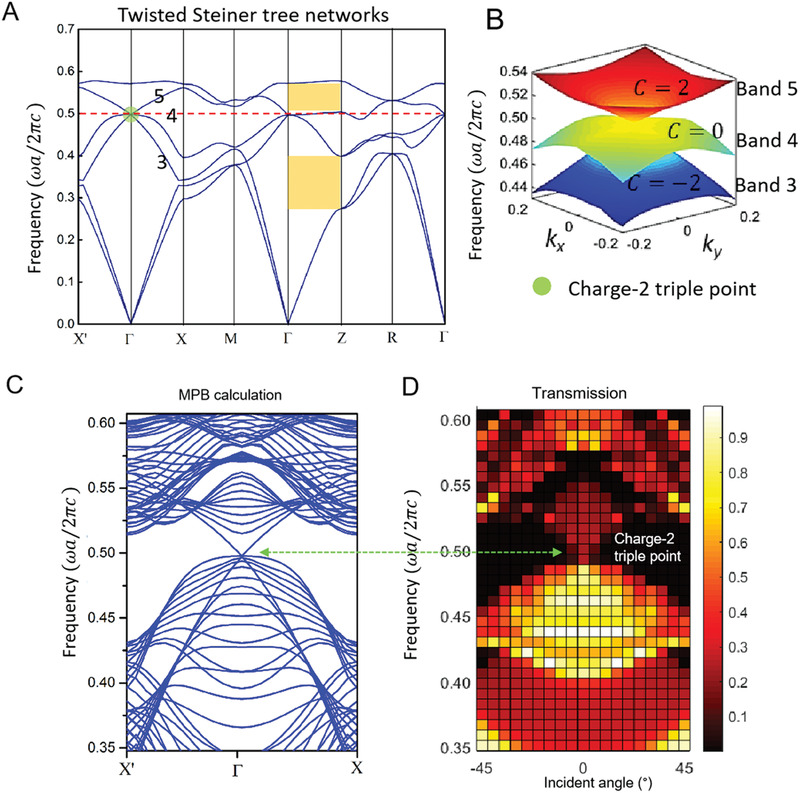
Twist induced topology transition in T‐STNs. a) Band structure of a T‐STNs with rod diameter 0.2*a*, and a dielectric permittivity *ε* of 13. The triply degenerate point at Γ is shown with a green sphere. Note that Bands 1&2, and Bands 3&4 along Γ‐Z are doubly degenerated. The stop gap along Γ‐Z direction are highlighted in yellow. b) Band structure in the vicinity of the triple point on Γ‐M‐X plane. Bands 3, 4, and 5 are represented in blue, green, and red, the Chern numbers (*C*) of ‐2, 0, and +2, respectively. And band 3 is TE modes, bands 4&5 are TM modes. c) Projected band structure along X’‐ Γ‐X direction. d) Simulated transmission spectra surrounding the triply degenerate point. The incident angle changes from −45° to 45° from Γ to M. The color bar indicates the normalized transmission at each point in momentum space.

The periodic dielectric structures of the STNs make them perfect candidates as 3D photonic crystals where energy band structures of photons can be formed.^[^ ^48^ ^]^ Unlike the photonic band structure of STNs (shown in Figure S10, Supporting Information), which lacks any upper band degeneracy at any of the high symmetry points, the T‐STNs possess a band degeneracy in the upper bands that provide a rare mechanism to the formation of a nontrivial Dirac‐like dispersion predicted by accidental degeneracy.^[^ ^30^ ^]^ The photonic band structure of T‐STNs (shown in **Figure** **4**a) shows a pronounced accidental triple degeneracy at the Γ point comprising two linear bands and a third flat band forming a Dirac‐like conical dispersion at *k*  =  0 and a frequency at *ωa*/2*πc*  =  0.497. This degeneracy of the three bands at the Γ point in T‐STNs emerges through a similar mechanism to the “twisted magic angle” effect in twistronics, such as bilayer graphene or 2D van der Waals materials,^[^ ^32^, ^33^, ^34^ ^]^ whose electrical conductivity strongly depends on the twisting angle and the layer distance. By tuning these two parameters, the coupling strength of the eigenmodes between adjacent layers through van der Waals interactions can be tuned and therefore influencing the electrical conductivity along the bilayer plane. Similarly, in STNs and T‐STNs metamaterial, the photonic property relies on the twisted symmetry change from STNs to T‐STNs, and the tuning of the coupling strength of the transverse electric (TE) and transverse magnetic (TM) modes in the k*_x_*−k*_y_* plane. Specifically, the 90° twist of STNs to T‐STNs gives rise to the symmetric conical shape of photonic band structure along X‐Γ‐X’ direction in the k*_x_*−k*_y_* plane (shown in Figures S10 and S11, Supporting Information). Meanwhile, by tuning the refractive index of the constituent material and the diameter of the rods, the photonic bands of TE and TM modes can be coupled to the same frequency at the Γ point through accidental degeneracy (shown in Figure S11 (Supporting Information), in which band 3 is TE modes, and bands 4&5 are TM modes). Therefore, STNs and T‐STNs metamaterial can be viewed as a unique 3D photonic counterpart to 2D van der Waals materials in twistronics, where the top/bottom six dielectric rods in T‐STNs can be regarded as the heterostructure in 2D van der Waals materials connected with a vertical dielectric rod,^[^ ^34^ ^]^ and the van der Waals interaction in T‐STNs is achieved through accidental degeneracy.

From a topological perspective, the Chern numbers of each band reveal the nontrivial nature of the triple degeneracy: the two linear bands possess Chern numbers of ± 2, while the flat band possesses a trivial Chern number of 0. A plot of the conical dispersion in the Γ − X − M plane is shown in Figure 4b, while a numerically calculated projected bulk band structure and spectral transmission map along Γ − X as a function of incident angle is shown in Figure 4c,d. In addition, the T‐STNs exhibit a clean stopgap along Γ − Z direction (shown in Figure 4a; and Figure S13c, Supporting Information).

The clean conical band structure in combination with a strong coupling strength of 60% at the triple point (The coupling strength as low as 1% in Weyl point photonic crystals.^[^ ^22^ ^]^) provides a platform for studying rare topological surface states. The stability of the degeneracy at the triple point is also studied based on a core‐cladding model, where the bands degeneracy can be preserved over a wide range of variations of coating parameters (detailed discussion in Section S10 and Figure S14, Supporting Information). We propose and discuss the experimental realization of the triple degeneracy point in STNs in Section S11 (Supporting information). It can also be envisioned that zero refractive index metamaterials made of dielectric materials can be realized in 3D STNs which may provide more freedom of design over zero‐refractive index metamaterials based on 2D structures.^[^ ^30^ ^]^

## 5 Conclusion and Outlook

We have investigated a new family of low‐density metamaterials inspired by the feature of the “shortest connection distance” in neurons: STNs. Using TPN, STNs nanostructures (smallest feature size of 200 nm) were fabricated with the lowest relative density compared to network structures designed from minimal surfaces, a property ensured by the use of the Steiner tree optimization approach. We demonstrated for the first time that the mechanical and topological properties of STNs can be triggered by a twisting degree‐of‐freedom. It is demonstrated that the twist leads to an enhancement of 20% of the mechanical strength in STNs and causes a transition from trivial to nontrivial topology of the photonic band structure in STNs. Further analysis showed that the twisted‐induced mechanical enhancement is a result of the strain energy redistribution between the top layer and the bottom layer along the z‐direction in the unit cell, and the topology transition results from the twist and the tuning of the coupling strength between the different photonic bands perpendicular to z‐direction through the accidental degeneracy. Our studies provide an avenue to create novel 3D low‐density metamaterials in other Bravais lattices and a route for twist‐angle‐controlled dispersion engineering and light‐matter interactions in 3D topological photonics, with potential applications for realization of 3D dielectric zero refractive index metamaterial for the mechanical robustness of STNs, given the use of two‐beam super‐resolution nanolithography,^[^ ^49^, ^50^ ^]^ and nanoscale coating techniques.^[^ ^24^ ^]^ Furthermore, the Steiner tree method could also become a platform for the discovery of exotic topological phenomena along with the recent development of inverse design,^[^ ^51^, ^52^ ^]^ and facilitate the development of 3D photonic neural networks research for its higher degree of connectivity.^[^ ^53^ ^]^

## 6 Experimental Section

### Fabrication of Steiner Tree Networks

The 3D STNs were fabricated using a custom‐built galvo‐dithered TPN system, that incorporates two galvo mirrors. The galvo mirrors expand the exposure in the lateral plane to compensate for the naturally elongated exposure in the longitudinal direction, thus providing cubically symmetric exposure and preserving the symmetry of the fabrication voxel. The photoresist was a custom developed hybrid organic/inorganic sol–gel described elsewhere.^[^ ^12^ ^]^ The sol–gel was prebaked at 75 °C for 30 min then exposed in the TPN system to fabricate 3D networks. The exposed resist was then immersed in a 50:50 solution of 1‐propanol:2‐propanol to dissolve the unexposed resist and reveal the freestanding networks in air. Further details can be found in Section S3 of the Supporting Information.

### Mechanical Property Characterization and Simulation

The mechanical characterization of Steiner tree networks was achieved using Hysitron TI 950 TriboIndenter. The simulation of the elastic properties of the structures was performed using finite element methods in ABAQUS. A detailed discussion can be found in Section S4 of the Supporting Information.

### Numerical Computation of the Photonic Band Structures

Full‐wave computations of the photonic band structures were performed with the open‐source package MIT Photonic Bands (MPB). The Bloch modes are calculated to be a 3D matrix of 64 × 64 × 64. Polarization and coupling strength analysis of the Bloch modes was performed via overlap integrals with linearly polarized plane waves in a similar manner to the chiral plane wave cases reported.^[^ ^13^ ^]^ Chern number analysis was performed by Takahiro's method which is detailed in Sections S6 and S7 in the Supporting Information.

## Conflict of Interest

The authors declare no conflict of interest.

## Supporting information

Supporting Information

Supplemental Movie 1

## Data Availability

The data that support the findings of this study are available from the corresponding author upon reasonable request.
