# Supplementary material for: Neuron‐Inspired Steiner Tree Networks for 3D Low‐Density Metastructures
Source: Adv Sci (Weinh). 2021 Aug 11;8(19):2100141. doi: 10.1002/advs.202100141 (PMC8498860; doi:10.1002/advs.202100141)
Supplement: Supplementary file 1 — Supporting Information [file ADVS-8-2100141-s001.pdf]

WILEY-VCH

© 2021 Wiley-VCH GmbH

ADVANCED SCIENCE
Open Access

Supporting Information

for *Adv. Sci.,* DOI: 10.1002/advs.202100141

Neuron-inspired Steiner tree networks for three-dimensional low-density metastructures

*Haoyi Yu, Qiming Zhang, Benjamin P. Cumming, Elena Goi, Jared H. Cole, Haitao Luan, Xi Chen, and Min Gu**

© 2021 Wiley-VCH GmbH

Supporting Information

Neuron-inspired Steiner tree networks for three-dimensional low-density metastructures

*Haoyi Yu, Qiming Zhang, Benjamin P. Cumming, Elena Goi, Jared H. Cole, Haitao Luan, Xi Chen, and Min Gu**

This PDF file includes:

Supporting Sections S1 to S11

Supporting Figures S1 to S14

Supporting Tables S1 to S5

Supporting text

Section S1 | Steiner tree method

The Steiner tree problem, originally proposed by Jacob Steiner,[1] is an umbrella term for a class of problems in combinational optimization. It is also named Steiner Minimal Tree (SMT), and has been widely studied with various conditions to interconnect for a given set of objects and predefined objective functions, therefore exists a variety of methods to solve.

A simplified method was repeated in this work based on the description by Christian Peter Klingenberg, following the original code presented by Warren D. Smith in 1992. As described by Warren D. Smith,[2] this algorithm implemented a branch-and-bound approach, where several assumptions and topology features are required to find the smallest connection distance for N sites in d-space (N is the number of connecting sites, and d is the dimension of the space)：

1. It contains N " sites" (x_1, x_2, ...x_N), and possibly K additional "Steiner nodes" (x_{N+1}, x_{N+2}, ...x_{N+k}).
2. Each Steiner node has valence 3, and the edges emanating from it lie in a plane and have mutual angle 120°.

3. Each regular site has valence between 1 and 3 (and generally ≤ 2).
4. 0≤ K≤ N-2.

It is easily proved that an SMT must be a Steiner tree and no angle of SMT is < 120°. Any three rays intersecting at mutual 120° angles must lie in a plane, hence Property 2 follows. Property 4 is a consequence of Properties 2 and 3.

Following these assumptions, we implement this algorithm on 8 sites in a simple cubic lattice. This simplified algorithm helps to search for the shortest connection distance for our proposed sites and generate the coordinates of the Steiner nodes. The gradient mapping results are shown in **Figure S1** left, where the connection distance is shown as a function of the position of the Steiner nodes. As described in reference [2], there is no direct solution for Steiner tree problems, calculation should be made based on assumptions such that the shortest connection distance can be achieved. For a simple cubic Bravais lattice, where symmetries make the situation easier, 6 secondary sites should be introduced for the calculation of the connection distance (shown in Fig S1). In addition, due to the symmetric nature of simple cubic, two types of secondary sites can be distinguished (type 1 and type 2), similar to the situation for two-dimensional honeycombs. Therefore, the total connection distance in a simple cubic lattice can be represented as a function of the positions of type 1 (x1, y1, z1) and type 2 (x2, y2, z2) secondary sites, in particular the z-positions. It is worthy to note that the computational effort increases very rapidly with the increasing numbers of data points.

Section S2 | Rigidity analysis of neuron-inspired Steiner tree networks

According to traditional 3D low-density structure theories, two main categories of geometries exist with different mechanical responses: stretching-dominated (rigid structures) and bending-dominated (non-rigid structures). As the most important concept in defining the mechanical behavior of low-density materials, a stretching-dominated structure is stiffer and stronger for a given mass, while a bending-dominated structure is relatively soft but absorbs energy better when compressed. To understand the rigidity of 3D Steiner tree networks

(STNs) and determine whether it is stretching-dominated or bending-dominated, a general principle, the Maxwell stability criterion, needs to be introduced.

The Maxwell stability criterion was introduced in 1864 by James Maxwell.[3] It describes the stability of a structure consisting of bars connected by pin-joints. The argument behind the Maxwell stability criterion is that, for a given structure with joints and rods, the rigidity depends on the kinematic constraints in dimensions or the connectivity between the struts.

As described in the Maxwell criterion, a structure with j frictionless joints and b rods subject to k kinematic constraints in d dimensions is considered. With these, a structure has the potential to be rigid if it satisfies the equation:

$$dj - b - k \leq 0 \begin{cases} d = 2 \; in \; 2D \\ d = 3 \; in \; 3D \end{cases} \qquad \text{(Eq. s1)}$$

This equation is a necessary but not sufficient condition to determine the rigidity of a low-density structure. To make the equation more accurate, structural mechanisms and states of self-stress must be accounted for. For a structure with *m* mechanisms and *s* states of self-stress, **Eq. s1** can be generalized to:

$$dj - b - k = m - s \qquad \text{(Eq. s2)}$$

A structure that is rigid will have m = 0, and a structure that is non-rigid will have m > 0. It is not possible to determine the number of mechanisms or states of self-stress from this equation, but if either is known, it is possible to determine the other.

In the case of the Steiner tree networks (STNs) and twisted Steiner tree networks (T-STNs), as we can see from **Eq. s2**:

$$dj - b - k = 16 > 0 \qquad \text{(Eq. s3)}$$

Our designed STNs are bending-dominated, corresponding to a non-rigid mechanical property. With this in mind, it is possible to assess the mechanical properties of STNs for experiment tests and numerical simulations.

There are four different kinds of biomimetic low-density networks that have been widely studied in low-density metamaterials, the octahedron, the octet-truss, the Kelvin foam lattice, the Gyroid lattice. Among these four different lattice structures, the octahedron and the octet-truss lattice structures are generally categorized as bending-dominated lattice structures. While Kelvin foam lattice and Gyroid lattice structures are defined as stretching-dominated lattice structures.[4]

The mechanical properties of low-density structures (or cellular solids discussed by M. F. Ashby.) rely on three factors:

(i) The properties of the solid of which the structure is made of (constituent material);

(ii) The topology and shape of the cell edges and faces; and

(iii) The relative density, $\bar{\rho}$, which is defined as the mass of the constituent material divided by the volume of the structures.

In the experiment, these mechanical properties of lattice structures rely on the relative density of fabricated structures given the condition that they are made of the same material. Mathematically, as Ashby discussed (2), the Young's modulus and the yield strength of the lattice structures follow a power scaling law with the relative density as,

$$E \sim B \times E_s \times \bar{\rho}^m \quad \text{(Eq. s4)}$$

$$\sigma \sim C \times \sigma_s \times \bar{\rho}^n \quad \text{(Eq. s5)}$$

in which, E_s and σ_s are the Young's Modulus and the yield strength of the constituent material, and the exponents m and n are defined by cell geometry. B and C are proportionality coefficients also defined the cell geometry. Here, we summarize the experimental results of these four lattice structures from the literature.[5]

As can be seen from **Table S1**, lattice structures with different geometries have different exponents in the power scaling law with relative density. STNs studied in this work showed similar scaling behaviour compared with Kelvin foam and Gyroid lattice, which proves that

STNs is dominated by the bending of the rods in terms of the scaling constants and proportionality constants.

A summary of the mathematical formulas of different lattice structures is shown in **Table S2**. Compared with the other four traditional lattice structures, STNs has the smallest relative density given the same unit size *a*, and rod diameter *D*.

Section S3. Two-photon nanolithography of 3D STNs

The fabrication of STNs with varied relative densities was achieved using a home-build galvo-dithering two-photon nanolithography (GD-TPN) system illustrated in **Figure S2**. Galvo-dithering gives us a smoother structure with a better circular-shaped cross-section. In addition, the use of GD-TPN also enables improvements in mechanical stability and fabrication feature size.

A series of 3D STNs are fabricated by tuning the fabrication conditions, such as laser power, writing speed, and galvo-dithering diameter. Our fabricated results are characterized by scanning electron microscope (SEM) to determine the rob diameter and structure size after the fabrication (shown in **Figure S3**). The relative density of our fabricated results is mathematically calculated out using the formula in **Table S2**. As we can see, each of our fabricated networks maintained very good structural integrity.

Section S4. Experimental measurement of 3D STNs using Nano-indentation

For the experimental characterization of 3D Steiner tree networks, Hysitron TI 950 TriboIndenter was used. The Hysitron TI 950 nanoindentation system incorporates the powerful measurement module and advanced control module, which dramatically enhances the accuracy of mechanical measurement, and provides unprecedented low-noise performance. As can be seen from **Figure S4.a**, a flat end probe from Bruker is used to test the whole structure's response towards a loading force shown in **Figure S4.b**. The loading force we used is defined as a function of time, with a loading speed of around $1000 - 2000\ \mu N/s$, which can accurately collect the mechanical responses from our samples. By collecting the

load-displacement/stress-strain signal from the Nano-indentation, the mechanical properties of the networks can be obtained.

The data obtained from lattice compression experiments carried out in this work has a broad range of stress-strain responses, and as such, it is important to formulate a consistent method to measure meaningful Young's modulus (E) and yield strength (σ) data. In every sample tested, the stress-strain data for each sample included a toe region, a linear region, and a failure region (**Figure S5.a**). The toe region is a non-linear segment of data at the beginning of loading, and it arises from slight misalignments and imperfections between the sample and the indenter probe. For each sample, the first section of stress-strain data was taken starting at the beginning of loading and going to the onset of failure. The maximum slope of this data subset is measured and taken to be the Young's modulus E. This is done to mitigate the effect of the toe region on the stiffness measurement. In our experiments, a line with slope E is taken with a 0.02% strain offset from the obtained Young's modulus fit, and the intersection of this line and the stress-strain data is taken to be the yield strength σ (**Figure S5.a**). All samples with the same relative densities are measured in nanoindentation at least three times; therefore, the deviations of the experimental results can be obtained. The deviations are calculated by taking one deviation in one group of data and normalizing it by its respective average Young's modulus or yield strength value. The resulting data can be found in **Tables S3** and **S4** and examples of stress-strain curves can be seen in **Figure S6**. The complete set of data are shown in **Figure S7**, and **Figure S8**. It is necessary to declare that the stress-strain curves are derived from the load-displacement curves directly measured from the nanoindentation.

Section S5. Finite element method simulation of the Young's modulus of 3D STNs

Finite element method (FEM) simulations of the uniaxial compression of 3D STNs were performed using software ABAQUS, which is a commercial finite element simulation software. Full-scale simulations of lattice structures will consume prohibitively computational memory, which is why our 5×1 cells subject to which we have chosen to simulate 5×1 cells

structures (shown in **Figure S9**) along with periodic boundary conditions (PBCs). The linear elastic solver in ABAQUS is chosen to model the Young's modulus of our networks. The structure was modeled using Solidworks to achieve a geometry that can precisely reflect that of the actual structure. A C3D10 10-nodes quadratic tetrahedral element was chosen to mesh the model during the simulation. Constraint boundary conditions were applied to the bottom surface of the structure (**Figure S9**).

1. For each node on X^--face: find a matching node on X^+-face.
2. Constraint in-plane displacements: $U_x^+ = U_x^-$, $U_y^+ = U_y^-$.
3. Repeat this process for Y faces.

Rotational degrees of freedom were not considered for solid structures. The Young's modulus of the simulated structure was defined as the ratio between the average strain and the average stress of the unit cell in the Z-direction, where F is the applied force on the structure and L is the height of the structure,

$$\mathrm{E} = \frac{F}{L\Delta z} \qquad \text{(Eq. s6)}$$

The Young's modulus of the material in our simulation is defined as 0.1 GPa for our polymer material, [6][7] and the poison ratio is defined as 0.3. The size of the designed unit in our simulation is 5 μm. It is important to note that, Abaqus is dimensionless simulation software, therefore, all values that were used in our FEM simulation are defined as proportional and consistent with each other. All simulated deformations are listed in **Table S5**.

Section S6. Photonic band structure of STNs

Full-wave computations of the photonic band structures were performed with the open-source package MIT Photonic Bands (MPB). The Bloch modes are calculated to be a 3D matrix of 64×64×64. The result is shown in **Figure S10**.

Section S7. Tuning band degeneracy through accidental degeneracy in T-STNs

The P$\bar{4}m2$ space group in which the T-STNs belongs is nonsymmorphic such that non-trivial band degeneracies at the high symmetry points would not normally be predicted to exist from the symmetry of the structure alone. Furthermore, structures in P$\bar{4}m2$ group are also achiral such that point degeneracies carrying topological charge such as Weyl points are also prohibited. Nonetheless, the T-STNs provides a rare mechanism for the formation of non-trivial Dirac-like point dispersions through accidental degeneracy.[8] Accidental degeneracy arises in an optical system in the presence of a specific set of geometric and optical parameters. The P$\bar{4}m2$ group is primitive tetragonal with mirror symmetry along the secondary and tertiary lattice directions. The T-STNs, therefore, possesses a square lattice in these two axes whilst also hosts an improper four-fold rotational symmetry along the principle lattice direction. Similar symmetries in 2D square lattices have induced double degeneracy between monopole (frequency ω_m) and dipole (frequency ω_d) eigenstates at the Γ point ($\boldsymbol{k} = 0$) that transition from quadratic to Dirac like linear dispersion when their frequencies coincide ($\omega_m = \omega_d$). The third band remains mostly flat through the degeneracy.

Accidental degeneracy results from the incompleteness of the Hamiltonian operator in a quantum system,[9] and it can be achieved by tuning the geometrical parameters in photonics systems.[10] Here, we show the band structures of T-STNs as photonic crystals (PCs) with different radius of the rods along X-Γ-X' direction. The band structure with accidental degeneracy and thus degeneracy point is shown in **Figures S11.b** and **S11.e**, which has a triply degenerate point at the Γ point at frequency f = 0.4897/ca. Here, the lattice constant is a, the diameter is D = 0.2a, relative permittivity ε =13, permeability μ =1, respectively. By carefully tuning the relative permittivity or the diameter of the cylinders, the band degeneracy point can be achieved. Therefore, the Dirac-like degeneracy point in T-STNs emerges as a consequence of accidental degeneracy at a particular combination of geometrical parameters.

Section S8. Numerical calculation topological charge of triply degenerate point in T-STNs

The topological charge of a degenerate point can be expressed as the summation of the first Chern numbers of the bands below the degenerate frequency.[11] However, the degeneracy of bands at the triple degeneracy point makes it difficult to calculate the Chern number of the bands using the traditional method, where the bands are separated, and no degeneracies or crossing exist between bands. To calculate the Chern numbers of the bands around the triply degenerate point, a discretized sphere in k-space needs to be built (shown in **Figures S12.a** and **S12.b**) to separate the bands degeneracy. On the two-dimensional surface of this sphere, a gap separates the three bands involved in the triple degeneracy. Numerically, it is convenient to use the gauge-invariant method from lattice gauge theory and demonstrated in the context of topological insulators by Fukui, Hatsugai and Suzuki.[12] We implemented this method in Matlab. For the convenience of the reader, we review the method in the following. Note that the Bloch eigenvectors used for calculation are obtained from MPB.

For discretized Brillouin zone in our case, the lattice points k_l $(l = 1, \ldots, N_1N_2)$ on the discrete Brillouin zone as

$$k_l = (k_{j1}, k_{j2}), k_{j_\mu} = \frac{2\pi j_\mu}{q_\mu N_\mu}, (j_\mu = 1, \ldots, j_\mu) \qquad \text{(Eq. s7)}$$

The Bloch wave function is periodic on the lattice

$$\left|n(k_l + N_\mu\hat{\mu})\right\rangle = |n(k_l)\rangle \qquad \text{(Eq. s8)}$$

where $\hat{\mu}$ is a vector in the direction with the magnitude $2\pi/q_\mu N_\mu$.

We require that $N_\mu = q_\nu N_B$ $(\mu \neq \nu)$ so that the unit plaquette is a square of the size (shown in **Figure S12**). Therefore, the link variable from the wave function of the *n*th band is defined as,

$$U_\mu(k_l) = \langle n(k_l)|n(k_l + \hat{\mu})\rangle / |\langle n(k_l)|n(k_l + \hat{\mu})\rangle| \qquad \text{(Eq. s9)}$$

The link variables are well defined, as long as $\langle n(k_l)|n(k_l + \hat{\mu})\rangle \neq 0$, which can always be assumed o be the case (one can avoid a singularity) by the infinitesimal shift of the lattice. And μ can only be 1 or 2, representing two different directions in k-space (shown in **Figure**

S12.c). From the link variable (**Eq. s9**), the field strength can be calculated using the four-link variables in one specific plaquette, and it can be defined as,

$$\tilde{F}_{12}(k_l) = \ln U_1(k_l)\, U_2\big(k_l + \hat{1}\big) U_1\big(k_l + \hat{2}\big)^{-1} U_2(k_l)^{-1} \quad \text{(Eq. s10)}$$

$$-\pi \leq \tilde{F}_{12}(k_l) \leq \pi \quad \text{(Eq. s11)}$$

Note that the filed strength is defined within the principal branch of the logarithm from equation (**Eq. s10**). Finally, the Chern number on the lattice, which is associated with the *n*th band as,

$$\tilde{c}_n = \frac{1}{2\pi i} \sum_l \tilde{F}_{12}(k_l) \quad \text{(Eq. s12)}$$

We perform this calculation process for all the squared plaquettes on the surface of the sphere using a spherical coordinate and sum up all the field strengths on this surface. The results are shown in **Figure S12.d**.

In our calculation, the radius of the sphere in k-space influences the calculations of the Chern numbers. This is because, at the edge of the Brillouin zone, the band will have crossings with other bands, which will generate singularity points in our integrals. As is shown in **Figure S12.d**, the Chern numbers we calculated are consistent even if we change the radius of the spheres. Therefore, the topological charge of the triple point in T-STNs equals the summation of the Chern numbers of band 1, band 2, band 3, and band 4, which is -2.

Section S9. Transmission properties of T-STNs

The polarization analysis of the bands using an overlap integral approach reveals the dispersion to be linearly polarized along $\Gamma - \mathrm{X}$ but unpolarized along the $\Gamma - \mathrm{M}$ directions.[13] The flat band becomes purely longitudinally polarized at the degeneracy but is linearly polarized elsewhere. As shown in **Figure S13**, a strong, linearly polarized stop gap is also observed along $\Gamma - \mathrm{Z}$ due to the anisotropy of the dielectric function along this axis.

Section S10. Stability analysis of the degeneracy at the triple point

The stability of the degeneracy at the triple point in T-STNs was performed on a core-cladding model that will be used in the experimental exploration of the applications of the triple point (the model is shown in Figure S14). The degeneracy frequency of the bands was plotted as a function of the permittivity, and the coating thickness of the coating material. As is shown in our results, the triple point can maintain its band degeneracy over a relatively wide range of variations of the parameters: a range of permittivity change: 6.75 to 13 (corresponding to Titanium Oxide and Silicon); a range of coating thickness change: 0.07a to 0.12a, and a range of normalized degeneracy frequency shift: 0.590 to 0.943.

This stability analysis of the degeneracy at the triple point gives us solid proof towards the experimental exploration of the applications (zero refractive index metamaterial) of the triple point in the future. For instance, given T-STNs with a unit size of 2 µm, and a coating permittivity of 13 (Silicon), the degeneracy of the bands maintain even if the coating thickness varies from 100 nm to 200 nm, with just a shifting of the degeneracy frequency.

Section S11. Proposal for experimental realization of triply degeneracy point in T-STNs

The triple point in T-STNs (a photonic crystal) belongs to a different physics regime compared with the triple point found in the phononic crystal,[14] therefore requires much more delicate physical conditions to realize. The phononic triple point in reference 14 solves the Hooke's law using acoustic waves, while the triple point in T-STNs is a solution to Maxwell’s equations for optical waves. This fundamental difference between optical waves and acoustic waves makes it quite challenging for the experimental realization of the triple point in T-STNs, where a high value of permittivity (refractive-index) of the constituent material is required. In addition, there exist very few experimental fabrication techniques to realize the high permittivity needed for the triple point in T-STNs.

Experimentally, 3D T-STNs polymer templates can be fabricated with designated sizes by TPN, as was achieved in this work for the measurement of the Young’s modulus and yield strength. However, the refractive index of most photosensitive polymers is insufficient to

create accidental degeneracy, and methods to increase the refractive index would be required. High refractive index double-inversion methods or high refractive-index sub-wavelength coating methods can be used to boost the refractive index.[15] These also provide a method to increase the mechanical strength of the structures, as is commonly done in the field of low-density metamaterials. The transmission properties of the 3D dielectric T-STNs can be measured using a typical transmission measurement system. By measuring the transmission spectrum along $\Gamma - X$ direction in the fabricated photonic crystal, the triple point can be mapped as indicated in the numerical simulations in **Figure 4.d** in the manuscript.

References:

[1] F. K. Hwang, D. S. Richards, *Networks* **1992**, *22*, 55.

[2] W. D. Smith, *Algorithmica* **1992**, *7*,137.

[3] J. C. Maxwell, *Philos. Mag.* **1864**, *27*, 250.

[4] M. F. Ashby, *Philos. Trans. Royal Soc. A* **2006**, *364*, 15.

[5] L. R. Meza, G. P. Phlipot, C. M. Portela, A. Maggi, L. C. Montemayor, A. Comella, J. R. Greer, *Acta Mater.* **2017**, *140*, 424.

[6] H. Yu, Q. Zhang, and M. Gu, *Opt. Express* **2018**, *26*, 32111.

[7] Y. Hu, Z. Lao, B. P. Cumming, D. Wu, J. Li, H. Liang, M. Gu, P*roc. Natl. Acad. Sci. U.S.A.* **2015**, *112*, 6876.

[8] X. Huang, Y. Lai, Z. H. Hang, H. Zheng, and C. T. Chan, *Nat. Mater.* **2011**, *10*, 582.

[9] B. Bradlyn, J. Cano, Z. Wang, M. G. Vergniory, C. Felser, Cava, R. J., Bernevig, B. A. *Science* **2016**, *353*, aaf5037.

[10] L. Xu, H. X. Wang, Y. D. Xu, H. Y. Chen, J. H. Jiang, *Opt. Express* **2016**, *24*, 18059.

[11] L. Lu, Z. Wang, D. Ye, L. Ran, L. Fu, J. D. Joannopoulos, M. Soljačić, *Science* **2015**, *349*, 622.

[12] T. Fukui, Y. Hatsugai, H. Suzuki, *J PHYS SOC JPN* **2005**, *74*, 1674.

[13] B. P. Cumming, G. E. Schröder-Turk, S. Debbarma, M. Gu, *Light Sci. Appl.* **2017**, *6*, e16192.

[14] Y. Yang, H. X. Sun, J. P.Xia, H. Xue, Z. Gao, Y. Ge, D. Jia, S.Q. Yuan, Y. Chong, B. Zhang, *Nat. Phys.* **2019**, *15*, 645.

[15] N. Tétreault, G. von Freymann, M. Deubel, M. Hermatschweiler, F. Pérez Willard, S. John, G. A. Ozin, *Adv. Mater.* **2006**, *18*, 457.

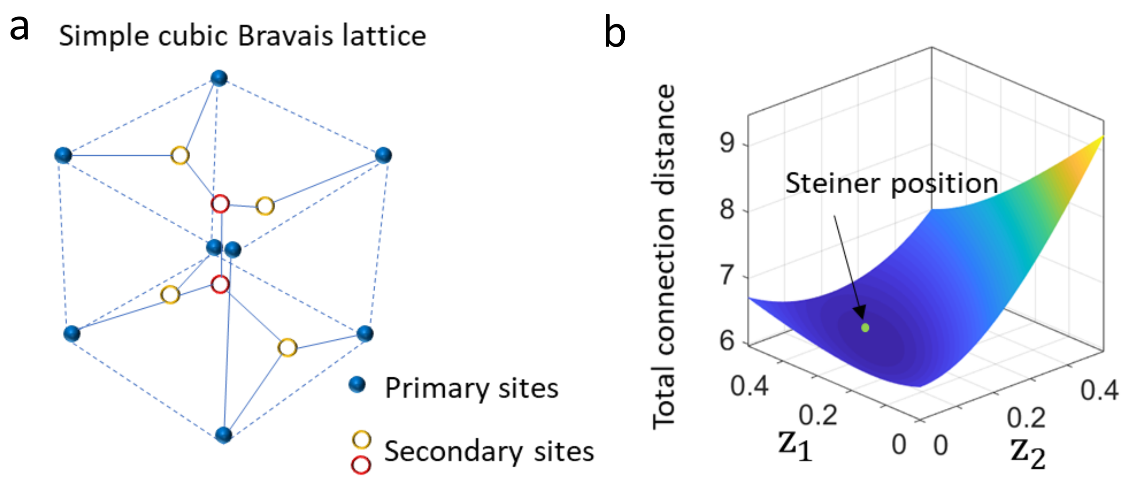

Figure S1. Steiner Tree method. (a) 3D SMT model in a simple cubic Bravais lattice. (b) Degradation mapping showing the total connection distance as a function of positions of the two secondary sites shown in Figure b. The Steiner position or the steiner nodes position is shown in (b).

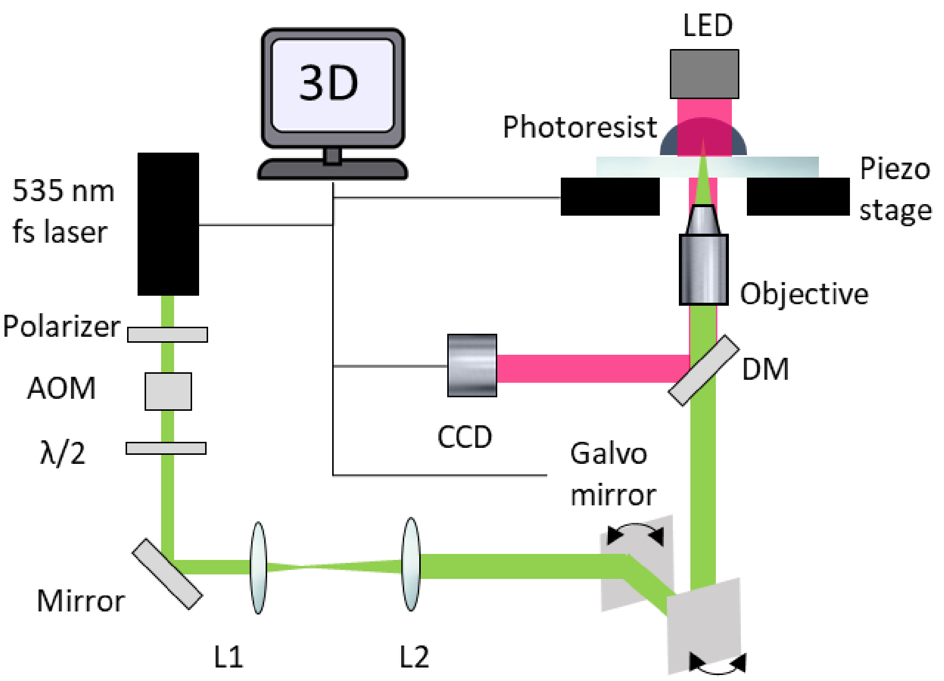

Figure S2. Galvo-dithered two-photon nanolithography system. A femtosecond laser beam operating at a wavelength of 535 nm (Coherent Fidelity + APE Harmonixx), with a pulse width of 270 fs and a repetition rate of 50 MHz, was steered by a combination of a 4f imaging system and 2D galvo mirrors (Thorlabs) into a 1.4 NA ×100 oil immersion objective (Olympus), and beam delivery and power are controlled by an acoustic optical modulator (AOM). L1 and L2 are focusing lenses, DM represents a dichroic mirror. Within the focus of the laser beam, polymerization occurs when the effective laser intensity is above the threshold of the photoresist. Micro-structures can be fabricated either by translation of the sample on the piezoelectric nanotranslation stage (Physik Instrumente) or the scanning of galvo mirrors.

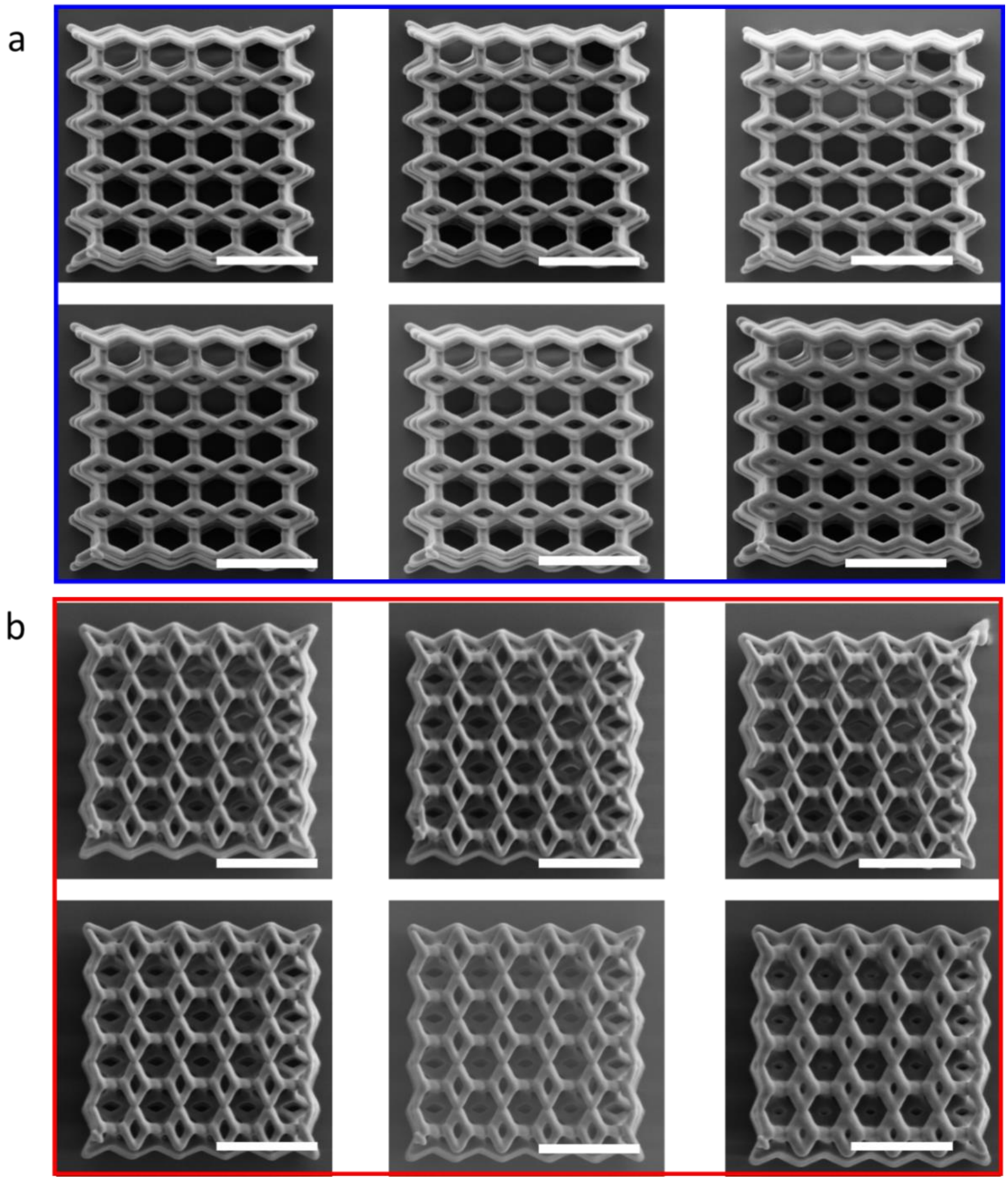

Figure S3. Scanning electron microscope (SEM) images of fabricated 3D STNs. (a). STNs fabricated by TPN. (b) SEM images of T-STNs fabricated by TPN. Rod diameters of our fabricated results range from 0.225, 0.288, 0.324, 0.346, 0.394, 0.431 µm (from left to right, top to bottom), and the unit cell is 2 µm. The scale bar is 4 µm.

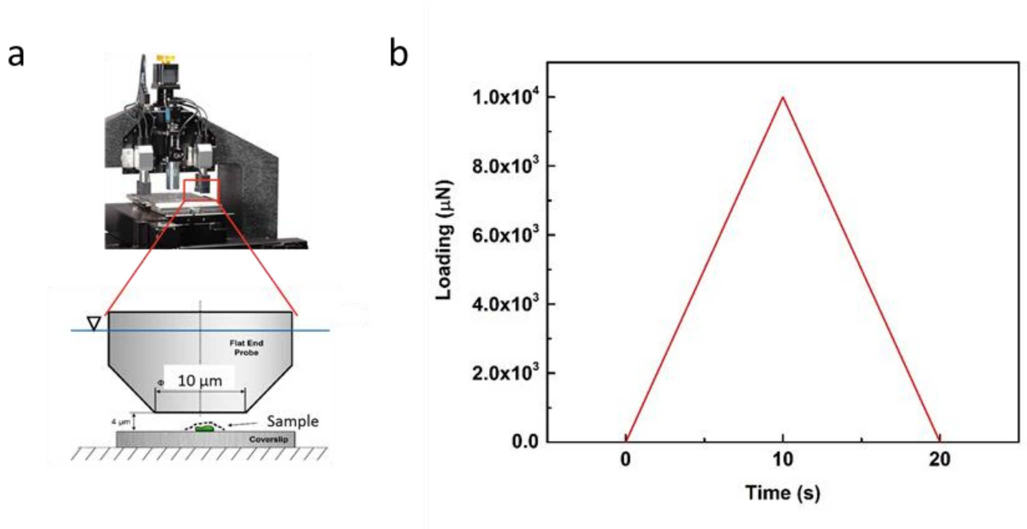

Figure S4. Illustration of the measurement of 3D STNs. (a) Experimental set-up of the measurement of STNs using a flat end probe. (b) Definition of the loading force as a function of time during the compression test used in Nano-indentation.

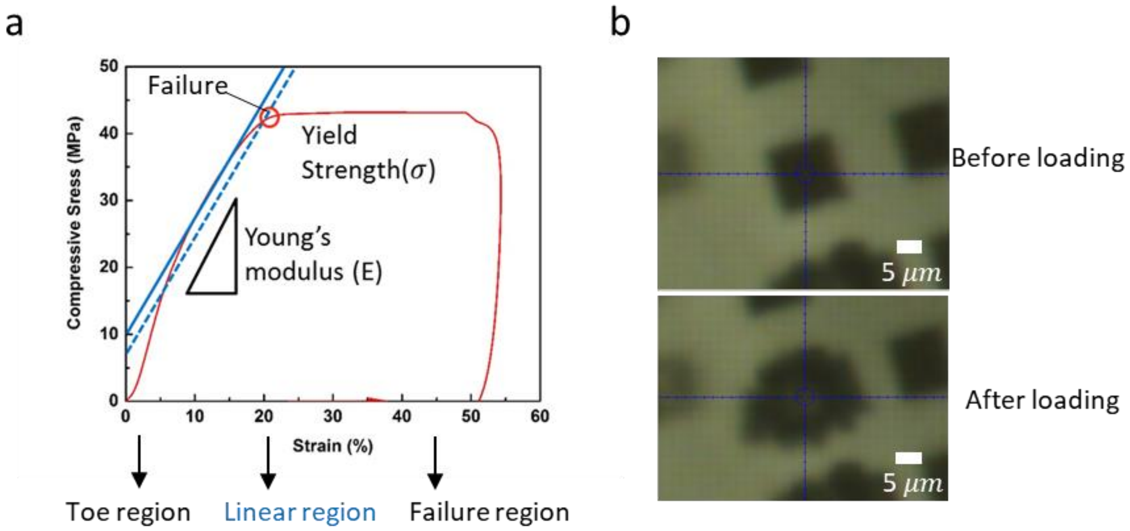

Figure S5. A typical compressive stress and strain curve obtained from nanoindentation. (a) The stress-strain curve obtained for bending dominated network structure. (b) Transmissive optical microscope images of networks before and after the compression, confirming the failure of mechanical integrity after exceeding the yield strength.

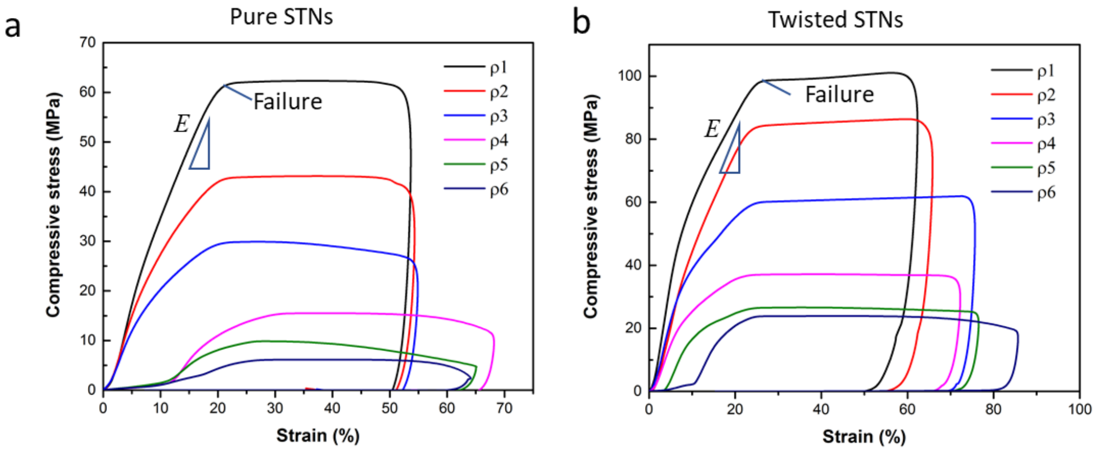

Figure S6. Experimental results of Compressive stress-strain curves of STNs and T- STNs with different relative densities ($\rho 1$- $\rho 6$ correspond to samples in Figures S2.a and S2.b).

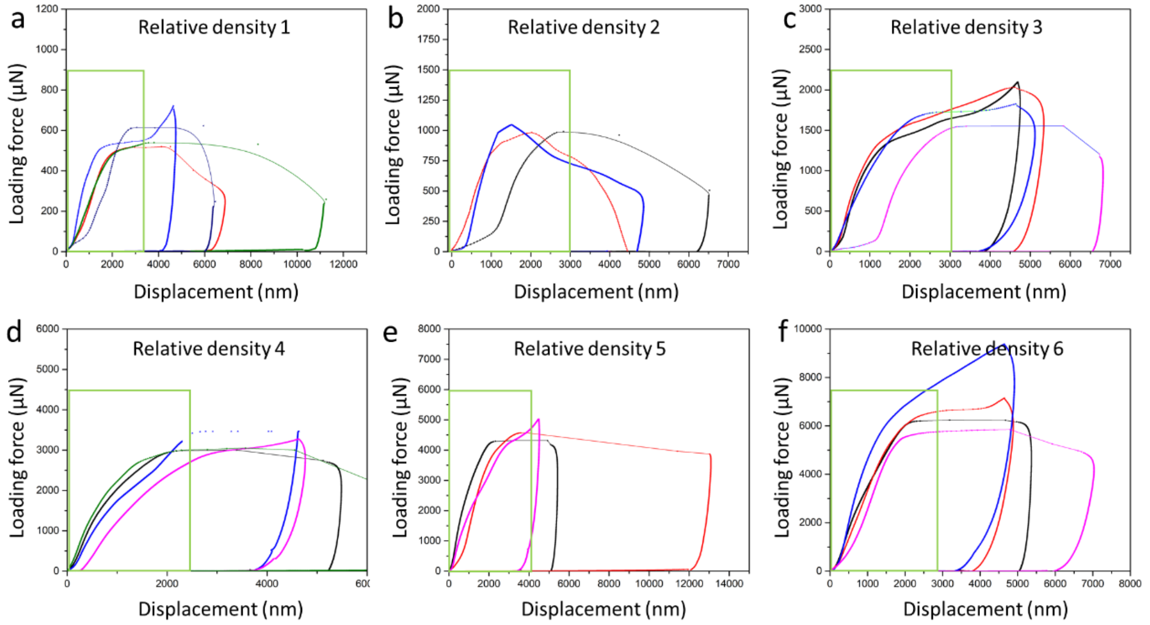

Figure S7. Load-Displacement curves of non-twisted Steiner tree microstructures obtained from compressive measurements. The compressive measurements are performed using the Bruker Hysitron nanoindentation. The measured samples are fabricated with 6 different relative densities (5.05%, 6.81%, 8.73%, 10.70%, 12.81%, 14.84% of the bulk material correspondingly, as described in the manuscript. Scanning Electron microscopic (SEM) images of the samples can be found in **Figure S2** in the Supporting information). 3-4 times of measurements (shown in different colors) were taken for samples with a certain relative density. All measurements share a great consistency and similarity during the elastic compression region (highlighted in green squares), where the Young's modulus and the yield strength of the microstructures can be derived based on the formula described in the manuscript and reference 18. Furthermore, after the compression process gets into the time when the microstructures collapsed (outside the green square region in the load-displacement curve), the probe of nanoindentation couldn't obtain the response from the collapsed structures, therefore the signals become unstable and vary drastically. Similar phenomenon can be found for twisted Steiner tree microstructures (**Figure S7**) as well.

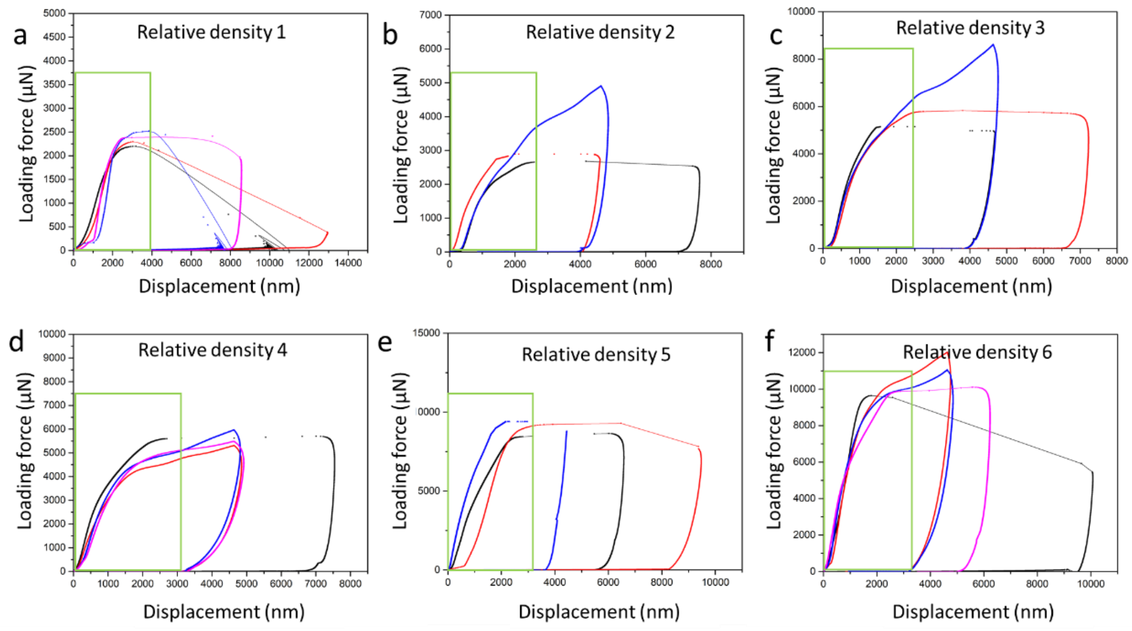

Figure S8. Load-Displacement curves of twisted Steiner tree microstructures obtained from compressive measurements. Each of the relative density corresponds to the relative densities discussed and analyzed in the manuscript.

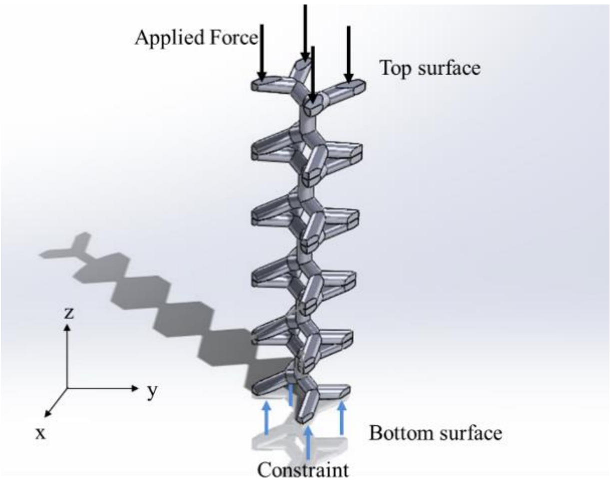

Figure S9. Simplified 5×1 unit structure modelled by Solidworks used in FEM.

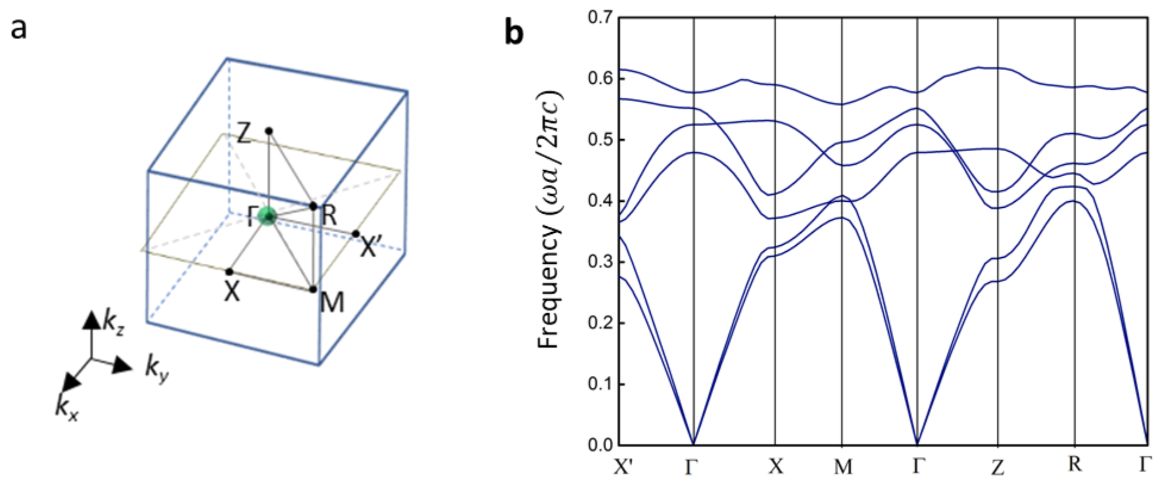

Figure S10. Photonic band structures of STNs. (a) Brillouin zone and high symmetry points. (b) photonic band structure of STNs calculated using MPB, with rod diameter 0.2*a*, and a dielectric permittivity ε of 13.

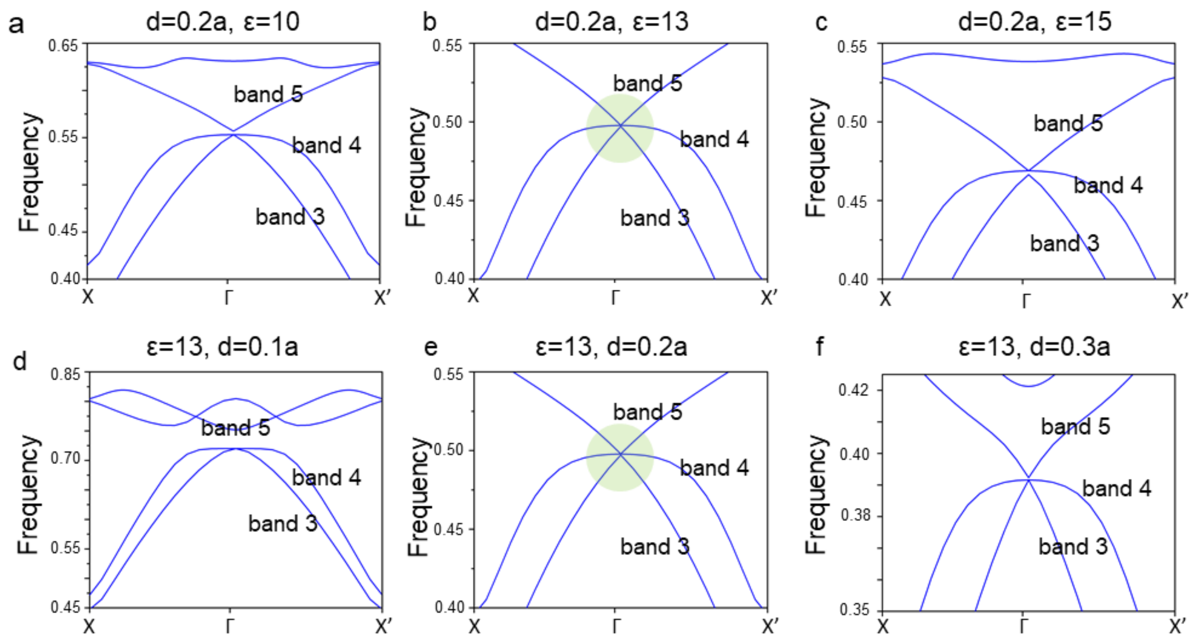

Figure S11. Effect of geometrical parameters on band structures of T- STNs. (a)-(c) Tuning the accidental degeneracy through changing the permittivity of the lattice. (d)-(f) Tuning the accidental degeneracy through changing the diameter of the lattice.

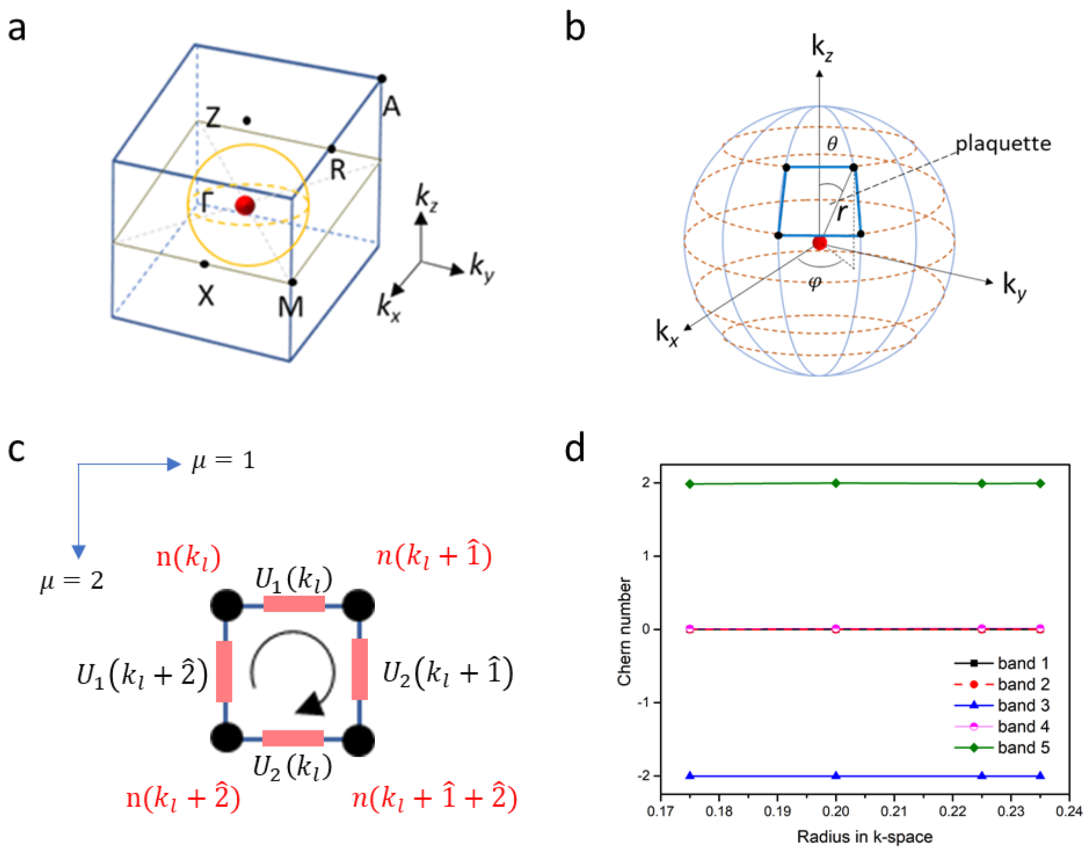

Figure S12. Calculation of the Chern numbers of bands based on Takahiro's method. (a) Brillouin zone. (b) A discretised sphere in k-space surrounding the triple point in a spherical coordinate system. A single plaquette is shown on the surface of the sphere. (c) Single plaquette demonstrating the link variables between each of the wave functions. The wave functions are represented as black dots, $n(k_l)$, $n(k_l + \hat{1})$, $n(k_l + \hat{2})$, and $n(k_l + \hat{1} + \hat{2})$.The link variables are represented as red blocks, $U_1(k_l)$, $U_2(k_l + \hat{1})$, $U_2(k_l)$, and $U_1(k_l + \hat{2})$. The direction to calculate the field strength for a specific plaquette is shown by an arrow. (d) Calculated results of Chern numbers for different bands as a function of the radius of the sphere in k-space. Band 1, band 2, and band 4 have the Chern number of 0, and band 3 and band 5 have the Chern number of -2 and 2 (see **Table S6** for detailed calculation results).

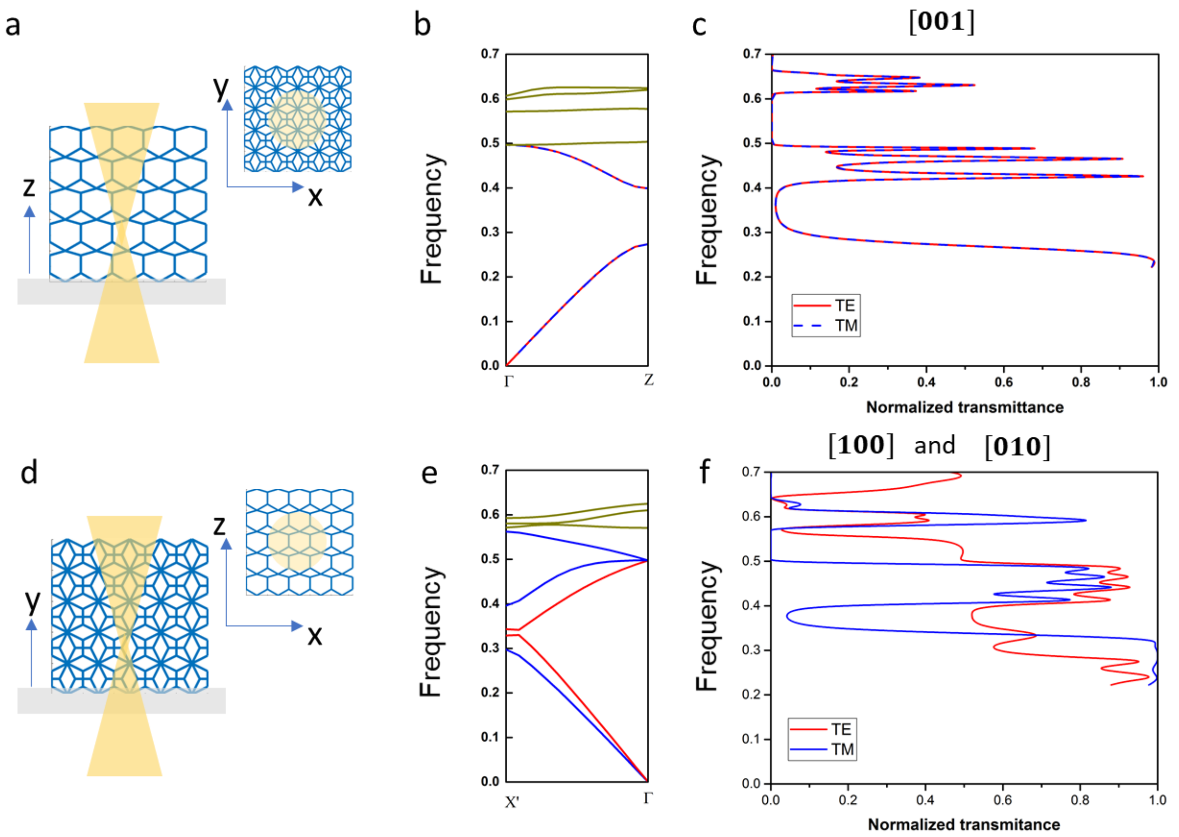

Figure S13. CST simulation of the transmission of T-STNs from z-direction and y-direction. (a) A cross sectional illustration of the simulation setup for the T-STNs clipped such that light is incident from the simulation input plane along the z-direction. (b) Band structure along Γ-X direction. (c) Transmission spectra of T-STNs from z-direction. (d) A cross sectional illustration of the simulation setup for the T-STNs clipped such that light is incident from the simulation input plane along the y-direction. (e) Band structure along X'-Γ direction. (f) Transmission spectra of T-STNs along y-direction. Transmission is measured at the simulation output plane. The simulation input and output planes are set a distance 2 μm away from the T-STNs x-y surfaces.

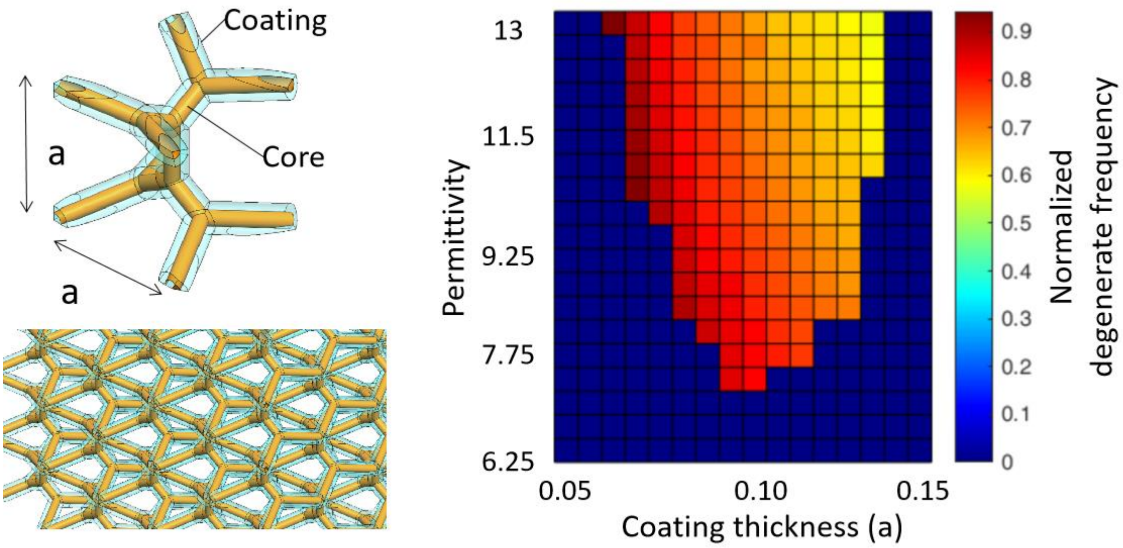

Figure S14. Stability analysis of the degeneracy at the triple point in T-STNs as a function of the coating thickness and the permittivity of the coating material. Left: CAD model of the core-cladding model used in the stability analysis (calculation step size for permittivity is 0.375, and the step size for coating thickness is 0.05a). Right: Stability of the triple point. The normalized degenerate frequency is plotted as a function of the coating thickness and permittivity. Color bar of the map shows the normalized degeneracy frequency of the triple point.

Table S1. Summary of the scaling constant (m and n) and the proportionality constants (B and C) of the Young's modulus and the yield strength scaling relationships respectively for each lattice structure made of solid polymer rods.

Rigidity	Lattice type	B	m	C	n
Rigid	Octahedron	1.090	1.830	1.590	1.920
Rigid	Octet-truss	0.820	1.770	1.310	1.880
Nonrigid	Kelvin foam	0.570	1.600	0.840	1.630
Nonrigid	Gyroid	0.319	2.000	0.315	1.500
Nonrigid	STNs	1.508	2.089	1.299	1.351
	T-STNs	1.290	1.903	2.308	1.656

Table S2. Formulas of relative densities and space groups for various lattice types

Lattice types	Space groups	Formula of relative density
8-points STNs	Pmmm, P$\bar{4}m2$	$(3\sqrt{3}+1)/4 \times \pi \times D^2/a^2$
Octahedron network	I$m3m$	$3\sqrt{2} \times \pi \times D^2/a^2$
Octet-truss network	I$m3m$	$3\sqrt{2} \times \pi \times D^2/a^2$
Kelvin foam network	F$m3m$	$(6\sqrt{2}-1) \times \pi \times D^2/a^2$
Gyroid network	I$4_1$32	$3\sqrt{2}/2 \times \pi \times D^2/a^2$

Table S3. Measured Young's modulus of 3D STNs

	STNs		T-STNs	
Relative density	Average Young's modulus (MPa)	Deviation	Average Young's modulus (MPa)	Deviation
0.0505	37.05	23.8%	43.14	25.6%
0.0681	43.12	16.3%	75.13	17.9%
0.0873	87.32	9.7%	127.05	21.5%
0.1070	168.70	10.3%	185.10	19.8%
0.1281	181.45	12.9%	250.11	15.5%
0.1484	287.72	11.6%	342.212	13.7%

Table S4. Measured yield strength of 3D STNs

	STNs		T-STNs	
Relative density	Average yield strength (MPa)	Deviation	Average yield strength (MPa)	Deviation
0.0505	4.47	29.1%	22.11	24.8%
0.0681	7.52	17.3%	26.31	16.4%
0.0873	14.02	12.6%	35.22	15.7%
0.1070	29.03	11.2%	58.83	22.3%
0.1281	41.37	18.7%	76.54	19.5%
0.1484	62.12	9.8%	98.64	11.9%

Table S5. FEM simulation results of Young’s modulus of 3D STNs

	STNs		T-STNs	
Relative density	Shape deformation	Young’s modulus (MPa)	Shape deformation	Young’s modulus (MPa)
0.0505	2.063	109.06	2.066	108.91
0.0681	1.841	122.22	1.698	132.51
0.0873	1.315	171.10	1.150	195.65
0.1070	1.265	177.87	1.116	201.61
0.1281	1.146	196.34	0.901	249.81
0.1484	0.861	261.32	0.642	350.41

Table S6. Calculation of Chern numbers of different bands in T-STNs.

Radius	Band 1	Band 2	Band 3	Band 4	Band 5
0.175	0.0007419	0.0010300	-2.0012287	0.0120709	1.9848153
0.200	0.0013700	0.0011900	-2.0014589	0.0145030	1.9973800
0.225	0.0011473	0.0010697	-2.0016823	0.0161738	1.9921410
0.235	0.0011473	0.0017423	-2.0017734	0.0168946	1.9941703
